# Commentary: Public Health System Perspective on Implementation of Evidence-Based Fall-Prevention Strategies for Older Adults

**DOI:** 10.3389/fpubh.2016.00252

**Published:** 2016-11-16

**Authors:** Tiffany E. Shubert, Matthew Lee Smith, Ellen C. Schneider, Ashley D. Wilson, Marcia G. Ory

**Affiliations:** ^1^Center for Health Promotion and Disease Prevention, University of North Carolina at Chapel Hill, Chapel Hill, NC, USA; ^2^South College, School of Physical Therapy, Knoxville, TN, USA; ^3^Department of Health Promotion and Behavior, The University of Georgia College of Public Health, Athens, GA, USA; ^4^Department of Health Promotion and Community Health Sciences, Texas A&M School of Public Health, College Station, TX, USA

**Keywords:** state health departments, evidence-based strategy, older adults, fall prevention, health promotion

## Background

Each year, approximately 30% of adults aged 65 years and older fall (1), resulting in significant morbidity, mortality, and decreased quality of life (2, 3). This problem is projected to increase as baby boomers age. Research confirms fall risk detection and evidence-based prevention programs offered in clinical and community settings that serve an aging population are effective at reducing the number of falls experienced (4, 5). To expand the reach of these services beyond the aging services network, the Centers for Disease Control and Prevention (CDC), the Administration for Community Living (ACL), and other funders are supporting opportunities for public health entities to become leaders in fall-prevention initiatives. The goal is to expand the infrastructure and entry points in both clinical and community settings to better meet the challenges of older adult fall risk management.

However, integrated community-clinical efforts integral to fall risk management are relatively new endeavors for State Departments of Health (DOH) (6). To be successful, DOH must recruit and engage a set of partners representing diverse sectors. Multi-sectorial collaborations are important for sustained adoption of evidence-based fall risk management practices. Such practices ensure the availability of a continuum of prevention and referral services for older adults.

This Commentary builds upon previous work from the State Falls Prevention Project (SFPP), a project funded by the CDC, in which DOH in New York, Colorado, and Oregon were charged with implementing clinical and community fall-prevention programs in specific geographic areas (6, 7). Now that the 5-year initiative has concluded, this Commentary reflects viewpoints of the SFPP Falls Evaluation and Technical Assistance (FETA) Team as guidance statements for future delivery of multi-level evidence-based fall-prevention interventions in the United States.

## State Falls Prevention Project

During the course of the SFPP, it became apparent the most effective implementation role for the DOH was to identify and connect health-care systems, community providers, and older adults to needed resources. Each DOH facilitated the implementation of three evidence-based fall-prevention programs, which were selected because of their ability to minimize risk of falling by improving balance, increasing strength, and providing education: (1) Tai Chi: moving for better balance; (2) stepping on; and (3) the Otago Exercise Program. Each state also developed strategies to increase clinical engagement in fall risk management through use of the CDC STEADI (STopping Elderly Accidents Deaths and Injuries) tool kit. Through this process, each DOH faced similar implementation challenges, which generated better appreciation of lessons learned from this experience and effective solutions.

## Challenges

During the first pilot year, the DOHs deployed the strategy of: (1) engaging with health-care providers through a traditional academic detailing model (i.e., provide lunch and a brief training session) to facilitate adoption of evidence-based fall risk management practices (8) and (2) working with community providers to increase access to community evidence-based fall-prevention programs (9–12). Several challenges were quickly realized by the entire SFFP team including:

1. Changing physician practice is a monumental task requiring the development of meaningful value propositions for each practice and ongoing relationship building, which could not be accomplished with a brief “lunch and learn” session.
2. Health-care organizations and providers (e.g., physicians, nurses, and physical therapists) typically have limited knowledge about value and availability of evidence-based fall-prevention programs available in the community.
3. There are many competing health-care and clinic efficiency initiatives that make it difficult for any new project to be viewed as a priority.
4. Each health-care system is unique. What motivates one system to embed fall risk management practices [i.e., modify Electronic Medical Records (EHR), adopt STEADI] will not necessarily be valued or motivating to other health-care systems in the same region.
5. There is widespread dissemination of evidence-based programs; however, a lack of program availability exists in many communities; few communities have a central source to provide a comprehensive, up-to-date list of available programs; this makes it challenging to schedule a patient in a timely manner.
6. Referral systems are fractured. No internal systems exist within a health-care system to refer a patient to a community-based program. The converse was true – no systems existed to connect an older adult identified as a fall risk by a community provider to a health-care provider.
7. There is a supply–demand dilemma – it is a challenge to build referrals from clinics to community programs (demand) while at the same time insuring you have enough programs in the community (supply).
8. It is important to identify potential partners interested in decreasing health-care costs and achieving better outcomes. However, not all partners will be ready to implement evidence-based programs as a cost-reducing measure.
9. Once a clinical-community linkage is created, long term sustainability of the linkage may be challenging due to personnel changes, program availability, and competing demands.

## Solutions and Lessons Learned

Reflecting on these challenges, the SFPP FETA Team, in collaboration with funders and grantees, gained perspectives about effective solutions. The role of the DOH as a “connector and convener” seemed the most effective model. As connector, the DOH educated and engaged stakeholders from health care and community settings about respective roles in fall-prevention efforts. As convener, the DOH brought stakeholders together to identify problems, discuss feasible strategies and solutions, and create state-specific systems to advance fall prevention. This strategy ultimately created stakeholder buy-in and ownership while developing potentially sustainable solutions to these challenges (6, 13). Table 1 presents lessons learned (with examples) from this project.

**Table 1:** **Lessons learned, with examples, from the State-driven Fall-Prevention Project from New York (NY), Colorado (CO), and Oregon (OR) Departments of Health (DOH)**.

Learned lesson	Description	Example
Dedicated staff time from DOH is required for relationship building	Substantial time is required to nurture and redefine (in some instances) pre-existing partnerships to the point where they are vested in implementing and sustaining change	Each DOH had established relationships with health-care systems through advisory boards and planning groups. Additional time required before partners valued and were ready to engage in practice change After committing to change, additional time was required to support/assist partners to complete implementation responsibilities.

Potential stakeholders have different goals and initiatives	Understanding market drivers for each stakeholder is an effective adoption and implementation strategy	All three states Provided tailored technical assistance to each partner Specifically addressed program alignment with business goals

Roles and responsibilities must be clearly defined	Effective fall risk management requires communication and collaboration between multiple partners Partners do not understand the parameters of their role. Gaps may exist in their management program	A large academic medical center adopted STEADI Planned to refer to evidence-based programs in the community Did not realize they needed to create a system to make those referrals happen

The DOH plays a role as a connector	The DOH can connect established and engaged partners with new partners by showcasing efforts of each	OR convened a “Health Systems Partner” meeting attended by five health-care systems, State Unit on Aging, AAA, DOH, and DHS Champions presented their STEADI model Key stakeholders presented their role in primary care fall risk management Many stakeholders had never met Many did not value partnering to manage fall risk Most health-care partners were unaware of DHS resources available to their patients The meeting resulted in A stronger connection and greater motivation to improve referrals among all players

Begin with early adopters or those in a high state of readiness	Highly motivated stakeholders due to market drivers or incentives or penalties are more willing to invest time and resources into effective partnerships	OR and CO Level -1 Trauma Centers are mandated to provide community injury prevention education Stepping on is one of the few evidence-based injury prevention programs target older adults The Level 1 Trauma Centers motivated to adopt and implement Stepping on In CO, the AAA were motivated to partner with the trauma centers for client referrals

		OR The rate of falls in a health system in Portland was putting it at risk of losing its Medicare 5-star rating. The health system was motivated to implement fall risk management solutions The DOH was able to connect the system with resources for health-care providers and community programs The system offers STEADI, the Otago Exercise Program, and refers to Tai Chi The Oregon Geriatric Education Center (OGEC) had identified falls and dementia as two priority areas They were willing to take on STEADI dissemination It aligned with research priorities OR is a Comprehensive Primary Care Initiative (CPCi) market OHSU Internal Medicine needed to meet CPCi quality standards OHSU was an early adopter of STEADI A large health-care system was not ready to implement a new fall-prevention program They had developed a fall risk management program It was not evidence-based DOH worked with them for over 3 years without success to implement evidence-based programs and/or refer system

Any new processes needs to fit within the clinical culture	Evidence-based practices to improve fall risk management will only be successful if the implementation process is simple fully integrated into the culture	NY developed a clinically-specific referral process Physicians were given a referral sheet with program contact information The referral sheet was provided to the patient OR aligned EBHP programs with the concept of a “specialist.” It is common for patients to receive referrals to a specialist Physicians and health-care organizations have specialty referral systems in place The EBHP *program* became a “specialist” Integrate referrals to EBHP into electronic medical records Salem Primary Care Clinic implemented a system which directly refers patients to physical therapists to implement the Otago Exercise Program

Celebrate successes, regardless of the size	Promote and publicize the accomplishments achieved by partners	NY made a video disseminated nationally about the success of STEADI implementation in one practice (https://youtu.be/XxDr4V06KaU)
		CO presented Level 1 Trauma Centers with a “Program of Excellence” award to publicly acknowledge accomplishments and reward efforts

Provide meaningful data to partners	Identify important drivers that influence your partners likelihood to change (i.e., cost, patient satisfaction)	CO Infographic of stepping on outcomes data More trauma centers have adopted the program

	Make sure data collected and analyzed is in alignment with drivers	NY Systematic evaluation of program processes and outcomes from physician practices implementing STEADI Clinic and provider-level STEADI reports to OHSU demonstrate improvements in claims billing and provider uptake

Identify innovative funding sources	Seeking out new and alternative partners can provide new referral and funding sources	OR – Tai Chi as a Medicare Part C Silver and Fit and Silver Sneakers FLEX now cover Tai Chi programs at the YMCA Similar options are being expanded in Silver Sneakers programs nationwide. CO – promoted to the Area Agencies on Aging EBHPs eligible for Older Americans Act Title IIID dollars

Plan for program sustainability from the beginning	Often grant-funded projects focus on number of programs started. This project focused maintaining and growing programs after funding	NY and OR Partners required to create sustainability plans Embed the EBHP into systems Promote systems change CO Focused on partners embedding the programs within stakeholder organizations Established a policy they would not provide subsidies for agencies or organizations to implement programs Offered mini-grants to cover start-up costs and facilitated instructor training The two major hospital systems hold the Stepping On licenses, cover all the costs of program implementation, and independently run the programs in their facilities

Leverage the infrastructure and lessons learned to pursue new fall-prevention funding opportunities	Build upon the strong foundation to continue to expand program reach	CO was awarded a grant by the Administration for Community Living to expand its falls prevention programing statewide
		NY was awarded a grant by ACL to develop new partnerships with Level 1 Trauma Centers to deliver EBHP across the state
		NY received additional state funds to implement fall risk management
		OHSU was awarded a grant to develop the STEADI toolkit for EHR dissemination with a national EHR company

*DOH, Departments of Health; DHS, Department of Human Services; AAA, Area Agencies on Aging; STEADI, Stopping Elderly Accidents, Deaths, and Injuries Tool; NY, New York; OR, Oregon; CO, Colorado; EHR, Electronic Health Record; OHSU, Oregon Health Sciences University; ACL, Administration for Community Living; EBHP, Evidence-Based Health Promotion Programs*.

The challenges and solutions inherent in implementation of fall-prevention initiatives served to define effective roles for DOH in these three states. Each DOH developed its own unique role in fall prevention; however, all the successful initiatives relied on DOH helping organizations identify the problem of falls and guiding them toward evidence-based solutions.

As federal and state agencies continue to fund delivery infrastructures to bring programs “to scale,” more effort should be given to defining the roles of each partner/stakeholder and connecting individual agencies to create/support a continuum of fall-prevention services.

## Author Contributions

All the authors were involved as evaluators of this 5-year initiative. All the authors wrote the manuscript and critically reviewed the manuscript.

## Conflict of Interest Statement

The authors declare that the research was conducted in the absence of any commercial or financial relationships that could be construed as a potential conflict of interest.
